# Antimicrobial consumption and impact of “Reserve antibiotic indent form” in an intensive care unit

**DOI:** 10.4103/0253-7613.70216

**Published:** 2010-10

**Authors:** Purabi Reang Sharma, Purabi Barman

**Affiliations:** Deparment of Clinical Pharmacology, Super Religare Laboratories, Fortis FLT LT Rajan Dhall Hospital, Vasant Kunj, New Delhi, India; 1 Microbiology, Super Religare Laboratories, Fortis FLT LT Rajan Dhall Hospital, Vasant Kunj, New Delhi, India

**Keywords:** Antimicrobial consumption, defined daily dose, reserve antimicrobial indent form

## Abstract

**Objective:**

To study the antimicrobial (AM) consumption, record the AM sensitivity pattern, and evaluate impact of “Reserve AM indent form” in the intensive care unit (ICU).

**Materials and Methods:**

The study was carried out in medical ICU over 4 months period at a tertiary care hospital. AM consumption was determined by defined daily dose (DDD) per 100 bed days for each month for consecutive 4 months. The average total AM consumption was calculated. The laboratory samples were processed, and the sensitivity pattern was determined. Some of the newer AM were categorised as “Reserve” and an indent form was made mandatory to be filled up prior to prescription.

**Results:**

The total AM consumption was 232 per 100 bed days. The commonly used AM were penicillin with β-lactamase inhibitor (21%) followed by antifungal drugs (13.4%), cephalosporins and macrolides (11.7%) each. The most common organism isolated was *Acinetobacter* (26.1%) followed by *Candida* (23.8%) and *Pseudomonas* (21.4%). The average occupancy index was 0.53, and the average duration of ICU stay was 6 days. The consumption of carbapenems (new AM) and antifungals decreased from 18.8/100 to 10.6/100 and 56.1/100 to 22.1/100 bed days, respectively, after the introduction of indent form.

**Conclusion:**

The “Reserve AM indent form” was helpful in reducing the AM consumption during the study period. The AM indent form can be used as an important tool to combat irrational use, AM resistance and can be implemented in AM stewardship programmes.

## Introduction

Antimicrobial (AM) resistance is an increasing problem in intensive care units (ICU). Infection with AM-resistant organisms can cause increased length of hospital stay, mortality, and costs to the patient.[1] Available data suggest that pattern of AM use influences the development of resistant organisms.[2] Widespread and excessive use of broad-spectrum AM, invasive medical devices, critically ill and immunosuppressed patients in ICU favour the spread of resistant organisms.[3,4] Hence, evaluation of drug utilization pattern in ICU along with information on the sensitivity pattern of microorganisms from time-to-time is very crucial. Studies on drug utilization contributes to rational drug use by describing drug use patterns, detecting early signals of irrational drug use and identifying interventions to improve drug use and follow-up. These studies also reveal quality of drug prescribing by important predetermined criteria including defined daily dose (DDD).[5] DDD is defined as the assumed average maintenance dose per day for a drug used for its main indication in adults. It provides a fixed unit of measurement, which is independent of price and formulation.[6] It is presented as numbers of DDDs/1000 inhabitants/day or, DDDs per 100 bed days for in-hospital drug use. This study was carried out with following objectives:

1. To evaluate the AM consumption pattern in the ICU.
2. To study the bacteriological profile and sensitivity pattern.
3. To evaluate the impact of “Reserve AM indent form” on AM consumption in the ICU.

## Materials and Methods

This study was carried out in ICU of a tertiary care hospital over a period of 4 months in 2008. A prospective analysis of all patients admitted in the ICU during study period was carried out. The AM prescribed and their dosages were noted on daily basis. Carbapenems, aztreonam, tigecycline, vancomycin, linezolid, teicoplanin, fluconazole, voriconazole, and amphotericin B were placed on restricted use, and a “Reserve AM indent form” [Figure 1] was introduced at the beginning of the study to observe its effect on the AM consumption. Prescribers were asked to fill the form before indenting any of the restricted AM with justification. DDD/ 100 bed days was calculated for different class of AM on monthly basis. The total AM consumption per month was calculated by adding all individual classes of AM. The consumption of reserve AM was calculated on monthly basis and compared at the end of 4 months.

**Figure 1:** Reserve Antibiotic Indent form

**Table:** 

**Reserved antibiotic list**: Carbapenems, Aztreonam, Vancomycin, linezolid, Teicoplanin, Fluconazole, Voriconazole and Amphotericin				
**Patient’s name/Age:**
IPID:	Ward:	Date of prescribing:
Provisional Diagnosis:
**Probable site of infection**: Bloodstream/ Respiratory/ Urinary/ Any other				
**Indication:**: Porphylactic/ Empirical/ Culture based		
**Name of Antibiotic prescribed**:		
**Justification**: (Kindly tick)		
	Prior antibiotic use
	ESBL Risk
	Immunocompromised
	Any other (Pls Specify)
**Signature/Department-**		

As per the ATC/DDD classification, the DDD/100 bed days was calculated as follows:

$$\mathrm{DDD}/100\;\mathrm{bed}\;\mathrm{day}\;=\;\frac{(\mathrm{Antibiotic}\;\mathrm{consumption}\;\mathrm{in}\;\mathrm{grams})\;\times \;100}{\mathrm{DDD}\;\times \;\mathrm{number}\;\mathrm{of}\;\mathrm{days}\;\times \;\mathrm{number}\;\mathrm{of}\;\mathrm{beds}\;\times \;\mathrm{occupancy}\;\mathrm{index}}$$

The occupancy index (OI) was calculated every month and was derived by dividing the number of occupied beds by the total number of beds in the ICU.

The microorganisms in the specimens were identified by standard biochemicals.[7] Sensitivity of the isolated organisms was carried out by Kirby Bauer method using CLSI guidelines.[8]

## Results

A total of 177 patients were admitted during the study period. Out of these, 128 patients were men and 49 women.

### Morbidity pattern

The common morbidity was pneumonia (47) followed by chronic obstructive pulmonary disease (45), sepsis with multiorgan dysfunction (25), and central nervous system disease (9). Out of the total pneumoniae cases, 22 were community acquired pneumoniae, 9 sepsis, 8 Type II respiratory failure, 5 interstitial lung disease, and 3 aspiration pneumoniae.

There were also 14 postsurgical and 4 trauma, 6 renal failure and enteric fever cases, whereas 7 patients with hepatitis. The remaining 14 patients had gastrointestinal tract infection, diabetic ketoacidosis, hypoglycemia, malignancy, etc.

### AM consumption

**Figure 2:**
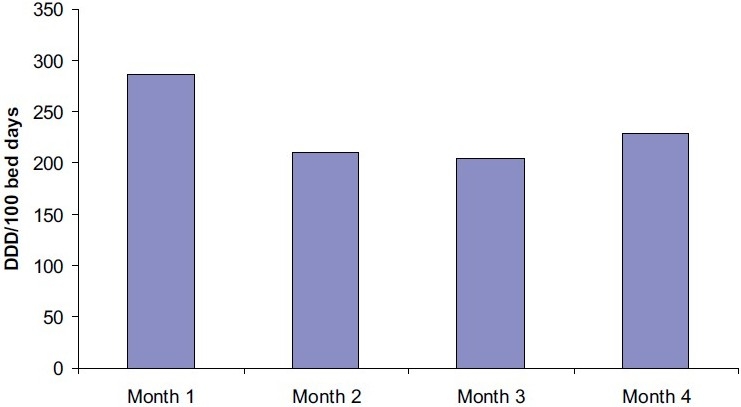
Total antibiotic consumption.

**Figure 3:**
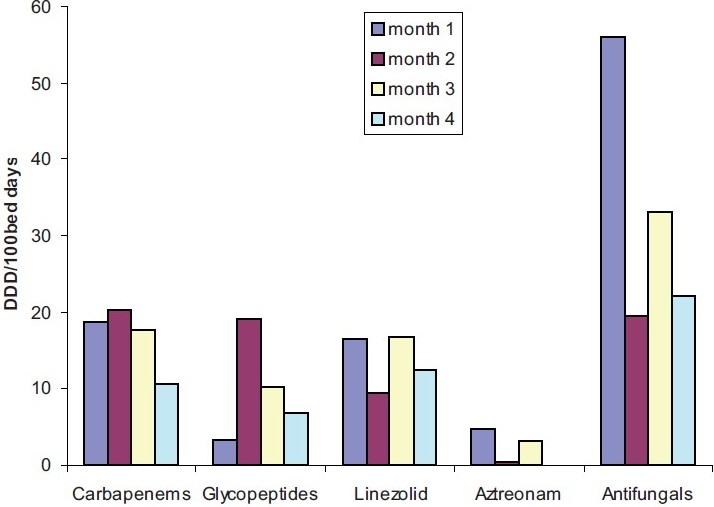
Trend of reserve antibiotics consumption.

The OI was 0.53, and average duration of hospital stay was 6 days. The monthly consumption of different class of AMs is shown in Table 1. The total AM consumption was 232/100 bed days. Penicillin+β-lactamase inhibitor was the most frequently used AM (21%), followed by antifungals (13.4%), cephalosporins and macrolides (11.7% each), and fluoroquinolone (10.4%). Multiple AM were prescribed in 152 (85.87%) patients. The frequency of use of newer AM was carbapenems (6.9%), linezolid (5.6%), and glycopeptides (4%). However, their consumption showed a reducing trend over the study period [Figure 2]. All patients were empirically started on broad-spectrum AM as per clinical symptoms. Positive bacteriological culture was observed in 24 patients, and AM were changed or continued as per the sensitivity report [Figure 3].

**Table 1:** Monthly AM consumption in intensive care unit

*AM*	*Month 1*	*Month 2*	*Month 3*	*Month 4*
Cephalosporins	36.5	41.95	23.7	12.4
Fluoroquinolones	26.25	28.5	27.2	17.9
Ampicillin/cloxacillin	2.2	6	9	7.4
Penicillin+β-	41.6	40.3	38.25	84.9
lactamase inhibitor				
Aminoglycosides	12.1	10.3	8.3	3.4
Macrolides	36.8	38.2	8.4	30.2
Carbapenems	18.8	20.3	17.8	10.6
Glycopeptide	3.4	19.2	10.2	7
Linezolid	16.5	9.5	16.8	12.5
Cotrimoxazole	9.7	0	0	0.3
Metronidazole	7.3	12.8	1.9	2.5
Clindamycin	6.7	3.7	2.2	12.5
Aztreonam	4.7	0.4	3.2	0
Antifungals	56.1	19.5	33.1	22.1

Values are expressed as DDD/100 beds; AM = Antimicrobial.

### Bacteriological profile and susceptibility pattern

The total numbers of samples received from 177 patients were 420, of which 227 was blood, 102 urine, 68 sputum, 13 pus/wound, and 10 body fluids. Bacterial growth was observed in 42 (10%) specimens with 42.8% sputum followed by blood (28.5%) and urine (19%).*Acinetobacter* was commonly isolated 11 (26.1%) followed by *Pseudomonas* 9 (21.4%) and yeast cells 10 (23.8%). Other organisms isolated were *Escherichia coli* 5 (11.9%), *Enterobacter* 2 (4.7%), Methicillin-resistant *Staphylococcus aureus* (MRSA) 1 (2.3%), and Coagulase negative *Staphylococcus* species (CoNS) 4 (9.5%). *Acinetobacter* was found to be multidrug-resistant and sensitive only to netilmicin in 45.5% isolates, whereas *Pseudomonas* was found to be sensitive to imipenem in 55.5% cases and meropenem in 44.44% isolates. *E. coli* was 100% sensitive to imepenem, meropenem, and netilmicin [Table 2].

**Table 2:** Sensitivity pattern of microorganisms (%) isolated from different specimens obtained from patients admitted in intensive care unit

	*Acinetobacter (n = 11)*	*Enterobacter (n = 2)*	*E. coli (n = 5)*	*Pseudomonas (n = 9)*	*Staphylococcus aureus (n = 1)*	*CoNS (n = 4)*
Amikacin	0	50	60	33.3	NT	NT
Amoxicillin+clavulanic acid	0	0	33.3	0	0	25
Ampicillin	0	0	0	0	0	0
Aztreonam	0	NT	NT	37.5	NT	NT
Cefepime	0	50	60	22.2	0	50
Cefoperazone	0	0	40	22.2	0	25
Ceftazidime	0	50	40	22.2	NT	NT
Ceftriaxone	0	50	33.3	33.3	0	25
Cefuroxime	0	0	33.3	0	0	25
Cephalothin	0	NT	–	0	0	25
Chloramphenicol	0	50	–	12.5	0	25
Ciprofloxacin	0	0	0	33.3	0	50
Gentamicin	0	0	40	25	0	50
Imipenem	0	100	100	55.6	NT	NT
Levofloxacin	0	0	0	33.3	0	100
Meropenem	0	50	100	44.4	NT	NT
Netilmicin	45.5	50	100	33.3	0	NT
Piperacillin+tazobactam	0	50	60	33.3	NT	NT
Ticarcillin+clavulanic acid	0	0	-	33.3	NT	NT
Tobramycin	0	50	0	22.2	NT	NT
Azithromycin	NT	NT	NT	NT	0	100
Cefaclor	NT	NT	NT	NT	0	100
Cefazolin	NT	NT	NT	NT	0	100
Clindamycin	NT	NT	NT	NT	0	100
Erythomycin	NT	NT	NT	NT	0	100
Linezolid	NT	NT	NT	NT	100	100
Oxacillin	NT	NT	NT	NT	0	25
Penicillin	NT	NT	NT	NT	0	0
Teicoplanin	NT	NT	NT	NT	100	100
Vancomycin	NT	NT	NT	NT	100	100

CoNS = Coagulase negative *Staphylococcus*; NT = not tested.

## Discussion

AM are among the most commonly prescribed drugs in hospitalized patients.[9] The emergence of AM resistance in ICU is of great concern as it attributes to poor prognosis, increases chances of drug interactions/side effects, prolongs the hospital stay, and increases cost of therapy. Monitoring the use of AM and review of sensitivity pattern are important. Development of AM resistance pattern is directly proportional to the volume of AM consumed. Therefore, to reduce the development of AM resistance regulation is essential.[10]

In this study, the AM consumption observed over 4 months period showed reducing trend, which may be due to the awareness created by the “Reserve AM indent form” or due to the seasonal variation in the disease profile and criticality of the patients. The AM consumption was 232/100 bed days which is comparable to the existing literature.[11,12]

Culture positivity was highest in respiratory samples; this can be due to the fact that the ICU was primarily a pulmonary unit. The commonest organism isolated was *Acinetobacter* suggest that most of the patients were critically ill, and many were transferred from other hospitals with multiple devices and prior broad-spectrum AM therapy.[13] This was followed by *Pseudomonas* and *Candida* responsible for nosocomial infections in ICUs.[14] The introduction of the reserve AM indent form reduced the consumption of newer AM as observed earlier.[15]

However, this study was of short duration with small sample size that may affect the validity of the conclusions drawn about AM resistance. Moreover, the seasonal variation also could not be taken into account, which has been shown to have a great impact on the disease profile and type of infections.[16,17] Although, this data gave a general overview of AM use in the ICU and also helped to find out the utilization pattern that would create awareness among the prescribers. This in turn would reduce inappropriate use of AM and bacterial resistance. Similar study of longer duration and with larger sample size would validate our observations and have an important bearing on AM stewardship programmes.
